# 6-Methyl-7,7,9-tripropargyl-7*H*-1,2,4-triazolo[4,3-*b*][1,2,4]triazepin-8(9*H*)-one

**DOI:** 10.1107/S1600536809031341

**Published:** 2009-08-15

**Authors:** Redwan Mohamed Zemama, Ibtissam Amari, Rachid Bouhfid, El Mokhtar Essassi, Seik Weng Ng

**Affiliations:** a Laboratoire de Chimie Organique Hétérocyclique, Pôle de compétences Pharmacochimie, Université Mohammed V-Agdal, BP 1014 Avenue Ibn Batout, Rabat, Morocco; b Institute of Nanomaterials and Nanotechnology, Avenue de l’Armée Royale, Madinat El Irfane, 10100 Rabat, Morocco; c Department of Chemistry, University of Malaya, 50603 Kuala Lumpur, Malaysia

## Abstract

The title compound, C_15_H_13_N_5_O, features a triazolyl ring fused with a seven-membered triazepinyl ring; the latter ring adopts a boat conformation (with the propargyl-bearing C atom as the prow and the fused-ring C/N atoms as the stern).

## Related literature

Triazepines are a class of compounds used in the treatment of neuronal disorders. They are also the reacta­nts for the synthesis of other heterocyclic compounds; see, for example: Essassi *et al.* (1977 ▶); Richter & Sheefelot (1991 ▶).
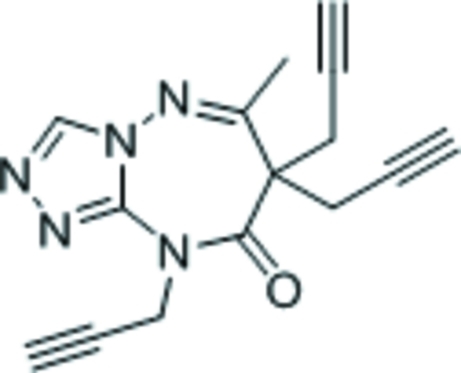


## Experimental

### 

#### Crystal data

C_15_H_13_N_5_O
*M* *_r_* = 279.30
Triclinic, 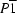

*a* = 7.6710 (2) Å
*b* = 8.2415 (2) Å
*c* = 12.9619 (3) Å
α = 108.510 (1)°
β = 90.659 (1)°
γ = 114.726 (1)°
*V* = 695.85 (3) Å^3^
*Z* = 2
Mo *K*α radiation
μ = 0.09 mm^−1^
*T* = 293 K
0.3 × 0.3 × 0.3 mm

#### Data collection

Bruker APEX2 diffractometer
Absorption correction: none
16879 measured reflections
3187 independent reflections
2577 reflections with *I* > 2σ(*I*)
*R*_int_ = 0.026

#### Refinement

*R*[*F* ^2^ > 2σ(*F* ^2^)] = 0.039
*wR*(*F* ^2^) = 0.129
*S* = 1.06
3187 reflections
191 parameters
H-atom parameters constrained
Δρ_max_ = 0.33 e Å^−3^
Δρ_min_ = −0.26 e Å^−3^

### 

Data collection: *APEX2* (Bruker, 2005 ▶); cell refinement: *SAINT* (Bruker, 2005 ▶); data reduction: *SAINT*; program(s) used to solve structure: *SHELXS97* (Sheldrick, 2008 ▶); program(s) used to refine structure: *SHELXL97* (Sheldrick, 2008 ▶); molecular graphics: *X-SEED* (Barbour, 2001 ▶); software used to prepare material for publication: *publCIF* (Westrip, 2009 ▶).

## Supplementary Material

Crystal structure: contains datablocks global, I. DOI: 10.1107/S1600536809031341/xu2584sup1.cif

Structure factors: contains datablocks I. DOI: 10.1107/S1600536809031341/xu2584Isup2.hkl

Additional supplementary materials:  crystallographic information; 3D view; checkCIF report

## Figures and Tables

**Fig. 1.**
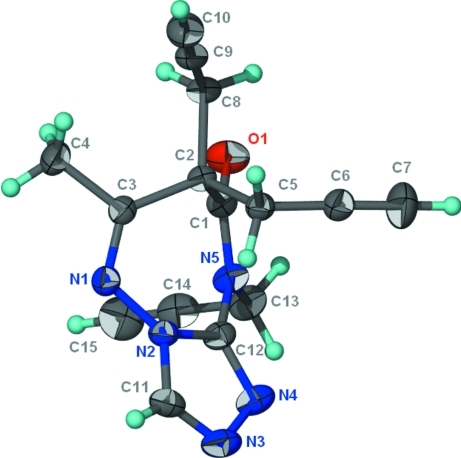
Thermal ellipsoid plot (Barbour, 2001) of C15H13N5O at the 50% probability level; hydrogen atoms are drawn as spheres of arbitrary radius.

## supplementary crystallographic information

### Experimental

To a solution of 6-methyl-7*H*-[1,2,4]triazolo[4,3-*b*][1,2,4]triazepin-8(9*H*)-one (1 g, 6 mmol) in *N*,*N*-dimethylformamide (20 ml), potassium carbonate (1.26 g, 9 mmol), propargyl bromide (0.7 ml, 9 mmol) and a catalytic amount of tetrabutyammonium broide were added. The mixture was stirred for 12 h. After the completion of the reaction (as monitored by TLC), the solid material was removed by filtration and the solvent evaporated under vacuum. Dichloromethane (20 ml) was added and the solution filtered. The solvent was removed and the product purified by column chromatography (30% ethyl acetate/hexane) to afford colorless crystals in 15% yield; m.p. 463 K. The formulation was established by proton and carbon-13 NMR spectroscopy in DMSO-*d*_6_.

### Refinement

Carbon-bound H-atoms were placed in calculated positions (C—H 0.93 to 0.97 Å) and were included in the refinement in the riding model approximation, with U_iso_(H) = 1.5U_eq_(C) for methyl H atoms and 1.2U_eq_(C) for the others.

### Crystal data

**Table:**

C_15_H_13_N_5_O	*Z* = 2
*M**_r_* = 279.30	*F*(000) = 292
Triclinic, *P*1	*D*_x_ = 1.333 Mg m^−^^3^
Hall symbol: -P 1	Mo *K*α radiation, λ = 0.71073 Å
*a* = 7.6710 (2) Å	Cell parameters from 6818 reflections
*b* = 8.2415 (2) Å	θ = 2.8–32.3°
*c* = 12.9619 (3) Å	µ = 0.09 mm^−^^1^
α = 108.510 (1)°	*T* = 293 K
β = 90.659 (1)°	Block, colorless
γ = 114.726 (1)°	0.3 × 0.3 × 0.3 mm
*V* = 695.85 (3) Å^3^	

### Data collection

**Table:**

Bruker APEX2 diffractometer	2577 reflections with *I* > 2σ(*I*)
Radiation source: fine-focus sealed tube	*R*_int_ = 0.026
graphite	θ_max_ = 27.5°, θ_min_ = 1.7°
φ and ω scans	*h* = −9→9
16879 measured reflections	*k* = −10→10
3187 independent reflections	*l* = −16→16

### Refinement

**Table:**

Refinement on *F*^2^	Primary atom site location: structure-invariant direct methods
Least-squares matrix: full	Secondary atom site location: difference Fourier map
*R*[*F*^2^ > 2σ(*F*^2^)] = 0.039	Hydrogen site location: inferred from neighbouring sites
*wR*(*F*^2^) = 0.129	H-atom parameters constrained
*S* = 1.06	*w* = 1/[σ^2^(*F*_o_^2^) + (0.0758*P*)^2^ + 0.0861*P*] where *P* = (*F*_o_^2^ + 2*F*_c_^2^)/3
3187 reflections	(Δ/σ)_max_ = 0.001
191 parameters	Δρ_max_ = 0.33 e Å^−^^3^
0 restraints	Δρ_min_ = −0.26 e Å^−^^3^

### Fractional atomic coordinates and isotropic or equivalent isotropic displacement parameters (Å^2^)

**Table:**

	*x*	*y*	*z*	*U*_iso_*/*U*_eq_	
O1	0.30213 (18)	0.47985 (14)	0.37303 (8)	0.0488 (3)	
N1	0.07241 (16)	−0.03916 (15)	0.11746 (9)	0.0346 (3)	
N2	0.22767 (16)	0.05768 (15)	0.07150 (8)	0.0323 (3)	
N3	0.4030 (2)	0.1070 (2)	−0.05735 (10)	0.0462 (3)	
N4	0.46123 (19)	0.28152 (18)	0.02864 (10)	0.0446 (3)	
N5	0.35151 (17)	0.38407 (15)	0.19840 (9)	0.0372 (3)	
C1	0.31447 (19)	0.35728 (17)	0.29631 (11)	0.0330 (3)	
C2	0.28282 (18)	0.16329 (17)	0.30330 (10)	0.0289 (3)	
C3	0.09422 (18)	0.01262 (17)	0.22312 (10)	0.0303 (3)	
C4	−0.0871 (2)	−0.0827 (2)	0.26582 (13)	0.0475 (4)	
H4A	−0.1893	−0.1748	0.2049	0.071*	
H4B	−0.1255	0.0114	0.3090	0.071*	
H4C	−0.0628	−0.1461	0.3109	0.071*	
C5	0.45567 (18)	0.11327 (18)	0.27425 (11)	0.0317 (3)	
H5A	0.4502	0.0191	0.3055	0.038*	
H5B	0.4400	0.0560	0.1947	0.038*	
C6	0.6465 (2)	0.2779 (2)	0.31450 (12)	0.0412 (3)	
C7	0.8004 (3)	0.4075 (3)	0.34405 (18)	0.0702 (5)	
H7	0.9233	0.5109	0.3676	0.084*	
C8	0.2658 (2)	0.18290 (19)	0.42552 (11)	0.0372 (3)	
H8A	0.3796	0.2944	0.4728	0.045*	
H8B	0.1525	0.2037	0.4431	0.045*	
C9	0.2492 (2)	0.0156 (2)	0.44969 (11)	0.0398 (3)	
C10	0.2456 (2)	−0.1166 (2)	0.46867 (14)	0.0512 (4)	
H10	0.2427	−0.2208	0.4836	0.061*	
C11	0.2649 (2)	−0.0213 (2)	−0.03028 (11)	0.0401 (3)	
H11	0.1996	−0.1499	−0.0742	0.048*	
C12	0.3531 (2)	0.24704 (19)	0.10322 (11)	0.0343 (3)	
C13	0.3765 (2)	0.56697 (19)	0.18940 (13)	0.0451 (4)	
H13A	0.4427	0.6700	0.2593	0.054*	
H13B	0.4564	0.5933	0.1338	0.054*	
C14	0.1885 (3)	0.5585 (2)	0.15971 (13)	0.0482 (4)	
C15	0.0358 (3)	0.5466 (3)	0.13320 (17)	0.0655 (5)	
H15	−0.0853	0.5372	0.1122	0.079*	

### Atomic displacement parameters (Å^2^)

**Table:**

	*U*^11^	*U*^22^	*U*^33^	*U*^12^	*U*^13^	*U*^23^
O1	0.0733 (8)	0.0385 (5)	0.0407 (6)	0.0331 (5)	0.0150 (5)	0.0102 (4)
N1	0.0306 (6)	0.0390 (6)	0.0356 (6)	0.0150 (5)	0.0058 (4)	0.0157 (5)
N2	0.0347 (6)	0.0390 (6)	0.0276 (5)	0.0190 (5)	0.0069 (4)	0.0138 (4)
N3	0.0575 (8)	0.0644 (8)	0.0371 (6)	0.0405 (7)	0.0202 (6)	0.0246 (6)
N4	0.0528 (8)	0.0531 (7)	0.0458 (7)	0.0315 (6)	0.0235 (6)	0.0285 (6)
N5	0.0478 (7)	0.0324 (5)	0.0395 (6)	0.0214 (5)	0.0155 (5)	0.0180 (5)
C1	0.0355 (7)	0.0307 (6)	0.0346 (7)	0.0165 (5)	0.0079 (5)	0.0114 (5)
C2	0.0318 (6)	0.0302 (6)	0.0278 (6)	0.0156 (5)	0.0082 (5)	0.0116 (5)
C3	0.0293 (6)	0.0325 (6)	0.0341 (7)	0.0158 (5)	0.0069 (5)	0.0152 (5)
C4	0.0324 (7)	0.0619 (9)	0.0511 (9)	0.0162 (7)	0.0116 (6)	0.0301 (8)
C5	0.0315 (6)	0.0366 (6)	0.0311 (6)	0.0180 (5)	0.0071 (5)	0.0132 (5)
C6	0.0358 (7)	0.0486 (8)	0.0410 (8)	0.0188 (6)	0.0084 (6)	0.0182 (6)
C7	0.0408 (10)	0.0661 (11)	0.0781 (13)	0.0050 (8)	0.0039 (9)	0.0197 (10)
C8	0.0454 (8)	0.0416 (7)	0.0290 (6)	0.0224 (6)	0.0112 (6)	0.0138 (5)
C9	0.0400 (7)	0.0508 (8)	0.0310 (7)	0.0195 (6)	0.0083 (6)	0.0183 (6)
C10	0.0515 (9)	0.0566 (9)	0.0538 (9)	0.0225 (8)	0.0074 (7)	0.0319 (8)
C11	0.0478 (8)	0.0517 (8)	0.0295 (7)	0.0313 (7)	0.0066 (6)	0.0129 (6)
C12	0.0390 (7)	0.0390 (7)	0.0361 (7)	0.0224 (6)	0.0122 (5)	0.0204 (6)
C13	0.0548 (9)	0.0323 (7)	0.0539 (9)	0.0186 (6)	0.0167 (7)	0.0236 (6)
C14	0.0677 (11)	0.0368 (7)	0.0506 (9)	0.0289 (7)	0.0161 (8)	0.0208 (7)
C15	0.0762 (13)	0.0636 (11)	0.0728 (12)	0.0435 (10)	0.0109 (10)	0.0274 (10)

### Geometric parameters (Å, °)

**Table:**

O1—C1	1.2094 (15)	C4—H4C	0.9600
N1—C3	1.2845 (17)	C5—C6	1.4583 (19)
N1—N2	1.3905 (15)	C5—H5A	0.9700
N2—C12	1.3630 (17)	C5—H5B	0.9700
N2—C11	1.3653 (17)	C6—C7	1.165 (2)
N3—C11	1.293 (2)	C7—H7	0.9300
N3—N4	1.3940 (18)	C8—C9	1.4643 (19)
N4—C12	1.3035 (17)	C8—H8A	0.9700
N5—C1	1.3690 (17)	C8—H8B	0.9700
N5—C12	1.3874 (17)	C9—C10	1.180 (2)
N5—C13	1.4808 (16)	C10—H10	0.9300
C1—C2	1.5463 (16)	C11—H11	0.9300
C2—C3	1.5357 (17)	C13—C14	1.455 (2)
C2—C8	1.5532 (17)	C13—H13A	0.9700
C2—C5	1.5620 (17)	C13—H13B	0.9700
C3—C4	1.4957 (19)	C14—C15	1.172 (3)
C4—H4A	0.9600	C15—H15	0.9300
C4—H4B	0.9600		
			
C3—N1—N2	117.64 (11)	C2—C5—H5A	108.8
C12—N2—C11	104.18 (11)	C6—C5—H5B	108.8
C12—N2—N1	130.86 (11)	C2—C5—H5B	108.8
C11—N2—N1	124.18 (11)	H5A—C5—H5B	107.7
C11—N3—N4	107.51 (11)	C7—C6—C5	178.18 (18)
C12—N4—N3	106.47 (12)	C6—C7—H7	180.0
C1—N5—C12	124.97 (10)	C9—C8—C2	113.42 (11)
C1—N5—C13	118.17 (11)	C9—C8—H8A	108.9
C12—N5—C13	116.66 (11)	C2—C8—H8A	108.9
O1—C1—N5	120.67 (11)	C9—C8—H8B	108.9
O1—C1—C2	121.75 (12)	C2—C8—H8B	108.9
N5—C1—C2	117.55 (10)	H8A—C8—H8B	107.7
C3—C2—C1	106.03 (10)	C10—C9—C8	176.60 (16)
C3—C2—C8	112.84 (10)	C9—C10—H10	180.0
C1—C2—C8	105.71 (10)	N3—C11—N2	110.81 (13)
C3—C2—C5	110.77 (10)	N3—C11—H11	124.6
C1—C2—C5	113.58 (10)	N2—C11—H11	124.6
C8—C2—C5	107.91 (10)	N4—C12—N2	111.01 (12)
N1—C3—C4	113.98 (12)	N4—C12—N5	125.45 (12)
N1—C3—C2	125.47 (11)	N2—C12—N5	123.34 (11)
C4—C3—C2	120.51 (11)	C14—C13—N5	110.59 (12)
C3—C4—H4A	109.5	C14—C13—H13A	109.5
C3—C4—H4B	109.5	N5—C13—H13A	109.5
H4A—C4—H4B	109.5	C14—C13—H13B	109.5
C3—C4—H4C	109.5	N5—C13—H13B	109.5
H4A—C4—H4C	109.5	H13A—C13—H13B	108.1
H4B—C4—H4C	109.5	C15—C14—C13	178.00 (18)
C6—C5—C2	113.64 (11)	C14—C15—H15	180.0
C6—C5—H5A	108.8		
			
C3—N1—N2—C12	−40.63 (18)	C1—C2—C5—C6	37.77 (15)
C3—N1—N2—C11	151.13 (13)	C8—C2—C5—C6	−79.06 (13)
C11—N3—N4—C12	0.01 (16)	C3—C2—C8—C9	68.72 (15)
C12—N5—C1—O1	−174.64 (13)	C1—C2—C8—C9	−175.83 (11)
C13—N5—C1—O1	0.2 (2)	C5—C2—C8—C9	−53.99 (15)
C12—N5—C1—C2	3.4 (2)	C2—C8—C9—C10	72 (3)
C13—N5—C1—C2	178.27 (11)	N4—N3—C11—N2	−0.81 (16)
O1—C1—C2—C3	112.39 (14)	C12—N2—C11—N3	1.25 (15)
N5—C1—C2—C3	−65.67 (14)	N1—N2—C11—N3	172.10 (11)
O1—C1—C2—C8	−7.63 (17)	N3—N4—C12—N2	0.80 (15)
N5—C1—C2—C8	174.31 (12)	N3—N4—C12—N5	−174.12 (12)
O1—C1—C2—C5	−125.74 (14)	C11—N2—C12—N4	−1.25 (15)
N5—C1—C2—C5	56.20 (15)	N1—N2—C12—N4	−171.23 (12)
N2—N1—C3—C4	173.10 (11)	C11—N2—C12—N5	173.80 (12)
N2—N1—C3—C2	−4.67 (18)	N1—N2—C12—N5	3.8 (2)
C1—C2—C3—N1	68.84 (15)	C1—N5—C12—N4	−148.84 (14)
C8—C2—C3—N1	−175.91 (12)	C13—N5—C12—N4	36.3 (2)
C5—C2—C3—N1	−54.80 (16)	C1—N5—C12—N2	36.8 (2)
C1—C2—C3—C4	−108.79 (13)	C13—N5—C12—N2	−138.05 (13)
C8—C2—C3—C4	6.46 (16)	C1—N5—C13—C14	−84.87 (17)
C5—C2—C3—C4	127.57 (13)	C12—N5—C13—C14	90.39 (15)
C3—C2—C5—C6	156.96 (11)		
